# Supplementary material for: Complex‐centric proteome profiling by SEC‐SWATH‐MS
Source: Mol Syst Biol. 2019 Jan 14;15(1):e8438. doi: 10.15252/msb.20188438 (PMC6346213; doi:10.15252/msb.20188438)
Supplement: Supplementary file 7 — Dataset EV6 [file MSB-15-e8438-s007.zip › feature_plots_bioplex/Q96IQ7.pdf]

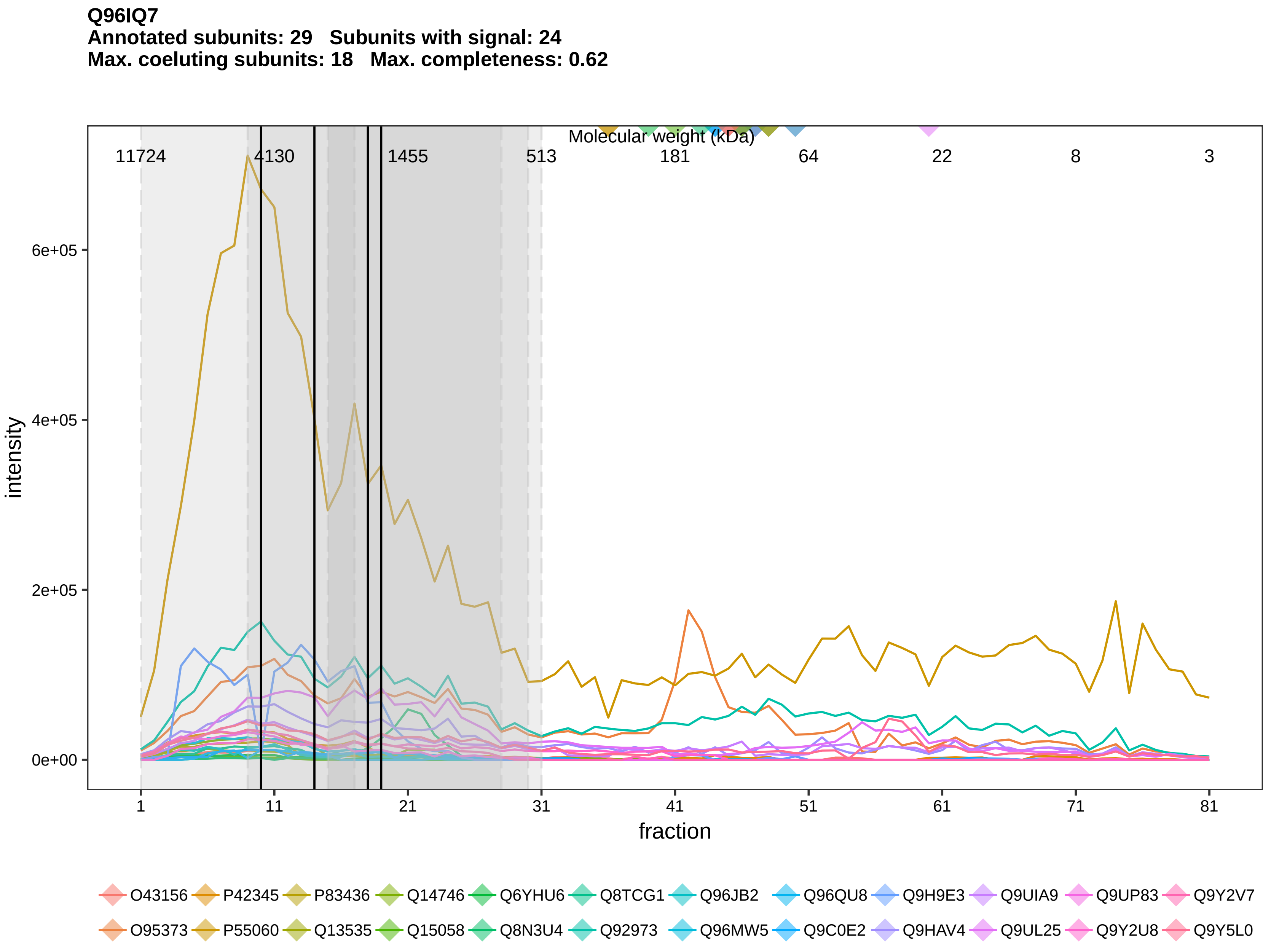

Q96IQ7
Annotated subunits: 29 Subunits with signal: 24
Max. coeluting subunits: 18 Max. completeness: 0.62
Molecular weight (kDa)
11724
4130
1455
513
181
64
22
8
3
intensity
6e+05
4e+05
2e+05
0e+00
fraction
1
11
21
31
41
51
61
71
81
O43156
P42345
P83436
Q14746
Q6YHU6
Q8TCG1
Q96JB2
Q96QU8
Q9H9E3
Q9UIA9
Q9UP83
Q9Y2V7
O95373
P55060
Q13535
Q15058
Q8N3U4
Q92973
Q96MW5
Q9C0E2
Q9HAV4
Q9UL25
Q9Y2U8
Q9Y5L0
